# Screen time and cardiorespiratory fitness in school-aged children: a sex-specific association independent of physical activity—evidence from Croatia

**DOI:** 10.3389/fpubh.2026.1928966

**Published:** 2026-08-21

**Authors:** Marko Badrić, Leona Roca Badrić, Ivana Nikolić, Dario Novak, Snježana Mraković

**Affiliations:** 1 Faculty of Teacher Education, University of Zagreb, Zagreb, Croatia; 2 Faculty of Kinesiology, University of Zagreb, Zagreb, Croatia

**Keywords:** 20 m shuttle run test, aerobic capacity, health, sedentary behavior, VO_2_ max

## Abstract

**Background:**

Excessive screen time has become an increasingly prevalent form of sedentary behavior among school-aged children and may adversely affect various health indicators, including cardiorespiratory fitness. However, the relationship between screen time and cardiorespiratory fitness remains inconsistent in the literature, particularly when overall physical activity is considered. This study aimed to examine the association between screen time and cardiorespiratory fitness in school-aged children while controlling for total physical activity and to determine whether sex differences exist in this relationship.

**Methods:**

A total of 306 school-aged children (163 girls, 143 boys; mean age 9.89 ± 0.60 years) participated in this cross-sectional study. Cardiorespiratory fitness was assessed using the 20 m shuttle run test, and maximal oxygen uptake (VO_2_max) was estimated accordingly. Screen time was assessed using a self-reported questionnaire and categorized into three groups (low, moderate, high). Total physical activity was assessed using the Physical Activity Questionnaire for Children (PAQ-C) and included as a covariate. A two-way analysis of covariance (ANCOVA) was conducted to examine the effects of sex and screen time on VO_2_max while controlling for physical activity.

**Results:**

Results indicate a significant main effect of screen time on VO_2_max after adjusting for total physical [F(2,299) = 6.40, *p* < 0.001, partial η^2^ = 0.04]. Children in the high screen time category had significantly lower adjusted VO_2_ max values than those in the low and moderate groups. Although no significant main effect of sex was observed, a significant interaction between sex and screen time was found [F(2,299) = 4.47, *p* = 0.01, partial η^2^ = 0.03]. Specifically, higher screen time was associated with lower cardiorespiratory fitness in boys, whereas no significant association was observed in girls.

**Conclusion:**

These findings suggest that excessive screen time may be associated with lower cardiorespiratory fitness in children, independent of overall physical activity levels, particularly among boys. These findings support public health strategies aimed at limiting excessive recreational screen time while promoting regular physical activity among children.

## 1 Introduction

In the contemporary era, the amount of time children spend in front of screens (TV, computers, mobile phones, and other digital devices) has increased significantly, which is associated with sedentary behavior, lower levels of physical activity (PA), and negative health outcomes. Multimedia content has become an integral part of children's everyday lives and plays a key role in their development (1). Watching television at home and using home computers are two of the main types of screen exposure among school-aged children (2). Over the past few decades, there has been an exponential increase in screen use among children and adolescents (3). Health guidelines recommend that children and adolescents aged 5 to 17 years should engage in at least 60 min of moderate-to-vigorous PA daily (4) and spend no more than 2 h per day in front of screens (5). However, with the emergence of the COVID-19 pandemic, the average screen time among children increased from more than 2 h per day before the pandemic to nearly 6 h per day during the initial phases of the pandemic, resulting in an overall increase of more than 3 h of daily screen time during the pandemic (6).

Various lifestyle factors influence PA status, and it is therefore important to investigate all related elements, including physical fitness, sedentary lifestyle, and obesity status (7). Promoting PA, limiting excessive screen time, and encouraging adequate sleep duration are important approaches for improving physical fitness (8). Research findings (9) showed that higher levels of PA were associated with cardiorespiratory fitness, regardless of compliance with screen-time guidelines, whereas screen time was a stronger predictor of children's weight status than PA. Higher levels of cardiorespiratory fitness may reduce the negative effects of excessive screen time (10), while an active lifestyle has been associated with lower screen exposure and higher cardiorespiratory fitness (11).

Cardiorespiratory fitness in children and adolescents has long been recognized as an important parameter for monitoring health, and in children, cardiorespiratory fitness is strongly associated with overall health status (12). Higher levels of cardiorespiratory fitness during childhood and adolescence are strongly associated with current health status and are also highly predictive of future health outcomes (13). Cardiorespiratory fitness largely depends on participation in PA. Furthermore, PA and excessive screen time have implications for cardiometabolic risk (14). A negative impact of high screen exposure and low cardiorespiratory fitness on both the current and long-term health of children and adolescents has been observed (15). There is a negative trend toward increased screen time, which is associated with reduced time spent outdoors and in moderate-to-vigorous PA among children (16). Previous cross-sectional studies have shown that cardiorespiratory fitness and screen time interact in their association with obesity-related parameters (17). Although previous studies have reported an inverse association between screen time and cardiorespiratory fitness, a longitudinal study involving 401 Brazilian children aged 7–15 years further demonstrated that prolonged screen time during childhood predicted lower cardiorespiratory fitness and increased waist circumference during adolescence (15). This indicates possible long-term consequences of sedentary behavior on physical fitness. A longitudinal study conducted on 175 Japanese adolescents aged 9–12 years showed that limiting excessive screen use is important for ensuring healthy development of cardiorespiratory fitness (18). Such trends suggest the development of inadequate lifestyle patterns already during primary school years.

Despite the existing literature examining cardiorespiratory fitness and screen time in relation to PA levels, the interaction between these factors remains insufficiently explored (14). This study is based on findings from previous research indicating a negative association between screen time, i.e., sedentary behavior, and cardiorespiratory fitness among school-aged children.

Although previous studies have demonstrated an inverse association between screen time and cardiorespiratory fitness in children, evidence remains limited regarding whether this relationship differs between boys and girls after adjustment for total physical activity, and findings across studies remain inconsistent (15, 18). Therefore, we extend existing research by examining the interaction between sex and screen time on cardiorespiratory fitness while controlling for total physical activity in school-aged children.

The aim of this study was to examine the interaction effect of sex and screen time on cardiorespiratory fitness in primary school children while controlling for physical activity levels. Based on the defined aim, two hypotheses were proposed: (1) there is an interaction effect of sex and screen time on VO_2_max, independent of physical activity; and (2) children who spend ≥4 h per day in front of screens have lower VO_2_max values compared to children with lower screen exposure.

## 2 Methods

### 2.1 Participants

This cross-sectional study included 306 primary school children from the continental region of the Republic of Croatia. Of the total sample, 163 participants were female and 143 were male. The mean age of the participants was 9.89 ± 0.60 years. Schools from three counties in the continental region of Croatia were randomly selected. A total of 12 primary schools were invited to participate, of which 10 agreed to participate (response rate: 83.3%). Before the study commenced, school principals were contacted by telephone or e-mail. Prior to data collection, permission for participation was obtained from the participating schools. Following institutional approval, written informed consent forms were distributed to parents or legal guardians. The study included only those children whose parents or legal guardians provided written informed consent and who personally signed the children assent form.

Children were eligible for participation if they regularly attended physical education classes, had written parental consent, and completed all anthropometric measurements, cardiorespiratory fitness testing, and questionnaire assessments used in the subsequent analyses. Health status was confirmed by classroom teachers based on their knowledge of the children and available information from school medical records, where applicable. Children with severe physical or psychological conditions that could interfere with safe participation in regular physical education classes or study procedures were not included. Children with overweight or obesity were not excluded from the study, provided they were able to participate in regular physical education classes.

All procedures conducted in the study were anonymous and carried out in accordance with the Declaration of Helsinki. The study was approved by the Ethics Committee of the Faculty of Teacher Education, University of Zagreb.

### 2.2 Research instruments

#### 2.2.1 Morphological measurements

Body height was measured using a portable high-precision stadiometer (Seca^®^ 213, Hamburg, Germany) with an accuracy of 0.1 cm. Body mass, body mass index (BMI), and body fat percentage were measured using the TANITA DC-360P dual-frequency body composition analyzer (Tanita Corporation, Japan), based on the principle of bioelectrical impedance. BMI was calculated as the ratio of body mass (kg) to the square of body height (m^2^). Waist and hip circumferences were measured using a measuring tape (19). Based on these measures, the waist-to-hip ratio (WHR index) was calculated as an indicator of body fat distribution.

#### 2.2.2 Measurement of cardiorespiratory fitness

Cardiorespiratory fitness was assessed using the 20-meter multistage shuttle run test (20mSRT). In this field-based test, participants ran back and forth between two lines set 20 meters apart. Running speed was regulated by audio signals and started at 8.5 km/h, increasing by 0.5 km/h every min. Each stage lasted approximately 60 sec, while the running pace (time interval between signals) was dictated by the audio signals (20). The aim of the test was for participants to maintain the required running pace for as long as possible. Maximal oxygen uptake (VO_2_max, mL·kg^−1^·min^−1^) was calculated using the following equation: VO_2_max = 31.025 + 3.238S – 3.248A + 0.1536 (A × S), where *S* represents the speed in kilometers per hour achieved at the end of the test, and *A* represents age in years (21). This equation is suitable for boys and girls aged 8 to 19 years. The test was terminated when the participant failed twice consecutively to maintain the required running pace or voluntarily stopped due to exhaustion. Testing was conducted in the sports halls of the participating schools during regular physical education classes, primarily in the morning. All measurements were performed by trained researchers following the same standardized testing protocol across all participating schools. Before testing, all participants received identical standardized instructions, and the original Léger 20-meter shuttle run protocol with standardized audio signals was used in every school. During the test, participants received standardized verbal encouragement from the researchers to ensure consistent motivation across testing sites.

#### 2.2.3 Measurement of physical activity

PA levels were assessed using the PAQ-C questionnaire (22). The questionnaire was designed for children aged 8 to 14 years, the purpose of which is to estimate the overall level of PA during the previous seven days. The questionnaire consists of nine items, and the overall PA score is calculated as the arithmetic mean of responses rated on a 5-point Likert scale, where 1 indicates low PA and 5 indicates high PA (23).

The Croatian version of the PAQ-C has previously been shown to be a reliable and valid instrument for assessing physical activity in school-aged children (24, 25). In the present study, the questionnaire demonstrated acceptable internal consistency (Cronbach's α = 0.799), consistent with the recommended threshold for research instruments (26) and indicating acceptable-to-good reliability (27). This value is also comparable with those reported in recent European validation studies, including the Italian version of the PAQ-C (28).

In addition, children estimated the amount of time they spent watching television and using the internet. The screen-time questions referred to participants' usual daily time spent watching television and using the internet. The questionnaire assessed overall daily screen time and did not distinguish between recreational and educational/school-related screen use. Screen time was assessed using a single-item self-report measure that has not been formally validated without an established reliability coefficient in the present sample. This represents an additional methodological limitation and is discussed further in Section 4.1. Using a self-report sociodemographic questionnaire, children provided information on sex, age, mode of transportation to school, and daily screen time (in hours). Screen time was categorized into three groups: 0–2 h/day (low level), 2–4 h/day (moderate level), and >4 h/day (high level). These categories were defined according to the international 24-h movement guidelines recommending no more than 2 hours/day of recreational screen time for school-aged children (11). The >4 h/day category was selected to represent prolonged recreational screen exposure substantially exceeding these recommendations and was consistent with previous studies investigating screen time and health outcomes in children (2, 29).

### 2.3 Statistical analysis

Basic descriptive statistics were calculated for continuous variables, including arithmetic mean, standard deviation, median, skewness, and kurtosis. Frequencies and percentages were calculated for categorical variables. Differences according to sex were examined using one-way analysis of variance (ANOVA) for all investigated parameters. To determine the significance of differences between subsamples according to sex and screen time in the dependent variable used to assess cardiorespiratory fitness, while controlling for the covariate of total PA, a two-way analysis of covariance (ANCOVA) was applied. To test the second study hypothesis, Bonferroni-adjusted pairwise comparisons of the adjusted estimated marginal means were performed following ANCOVA. Specifically, the high screen-time group (>4 h/day) was compared with the low (0–2 h/day) and moderate (2–4 h/day) screen-time groups. Effect sizes were expressed as partial eta squared (η^2^), and 95% confidence intervals were reported for the pairwise comparisons. The magnitude of partial η^2^ was interpreted according to conventional benchmarks, with values of 0.01, 0.06, and 0.14 indicating small, medium, and large effects, respectively.

Regarding ANCOVA assumptions, the normality of residual distribution was tested using the Shapiro-Wilk test. Visual inspection of the Q-Q plot did not indicate significant deviations from normal distribution, confirming acceptable residual normality. Levene's test for homogeneity of variances yielded *p* = 0.315, indicating no significant differences in variances between groups and confirming that the assumption of homogeneity of variance was met. Analysis of linearity between the covariate and the dependent variable confirmed the existence of a linear relationship, thereby satisfying the ANCOVA assumption. Furthermore, the assumption of homogeneity of regression slopes was met, as no statistically significant interaction between the covariate and the factors was observed. Potential influential observations were evaluated using Cook's distance together with standardized and studentized residuals. To assess the robustness of the findings, sensitivity analyses were performed by excluding the observations with the highest Cook's distance values and repeating the ANCOVA model. Statistical analyses were performed using IBM SPSS Statistics software, version 29.0. The level of statistical significance was set at *p* < 0.05.

## 3 Results

Table 1 presents the distribution of children according to screen time categories (television, computer, and mobile devices). The largest proportion of participants belonged to the moderate category (51.6%), i.e., the group spending between 2 and 4 h per day in front of screens. The high screen-time category included 21.6% of participants, indicating that approximately every fifth child belonged to the group with high screen exposure. The distribution of participants across screen-time categories did not differ significantly between boys and girls [χ^2^(2) = 4.97, *p* = 0.083], indicating that boys were not more likely than girls to belong to the high screen-time category.

**Table 1:** Number of participants according to screen time.

Screen	*N*	%
1 Low screen time	82	26.8
2 Average screen time	158	51.6
3 High screen time	66	21.6
Total	306	100.0

The results of descriptive statistics for the total sample are presented in Table 2. The average age of the children was 9.89 ± 0.60 years. Values for body mass index (M = 18.83 ± 4.20) and body fat percentage (M = 20.74 ± 8.88) indicated variability within the sample. Positive skewness was observed in the distribution of body mass (skewness = 1.09). The average VO_2_max value was 41.14 ± 1.99 mL·kg^−1^·min^−1^. Total PA showed a moderate average value (M = 2.96 ± 0.72). Children spent more time on the internet on average (M = 1.36 h) than watching television (M = 1.09 h). Skewness and kurtosis values for all variables were within the ±1.5 range.

**Table 2:** Descriptive indicators of morphological characteristics, physical fitness, physical activity, and screen time in the total sample of children (*N* = 306).

Variable	M	SD	Min	Max	Skew	Kurt
Age	9.89	0.60	8	12	0.04	0.40
Body height (cm)	145.31	8.23	124.00	168.00	−0.08	−0.17
Body weight (kg)	40.56	11.96	22.50	85.60	1.09	0.90
Fat mass %	20.74	8.88	3.0	43.1	0.59	−0.28
BMI	18.83	4.20	10.8	34.2	1.11	0.93
WC (cm)	66.14	10.44	35.0	98.0	0.61	0.41
HC (cm)	78.69	10.34	52.0	116.0	0.65	0.47
WHR	0.84	0.06	0.61	0.99	−0.51	1.23
VO_2_ max	41.14	1.99	35.24	45.69	−0.12	−0.11
Physical activity	2.96	0.72	0.73	4.71	0.08	−0.30
TV time (hours)	1.09	0.94	0.00	3.00	0.54	−0.57
Internet time (hours)	1.36	1.10	0.00	4.00	0.24	−1.25

M, mean; SD, standard deviation; Min, minimum values; Max, maximum values; SKEW, skewness; KURT, kurtosis; WC, waist circumference, HC, hip circumference; VO_2_ max, Maximum oxygen uptake; WHR, Waist-to-Hip Ratio.

Table 3 presents the results of the one-way analysis of variance (ANOVA) used to determine differences between boys and girls in morphological characteristics, physical fitness indicators, and patterns of PA and screen time. The analysis revealed no statistically significant sex differences in age [F(1, 304) = 0.56, *p* = 0.46, η^2^ = 0.00]. Boys had significantly greater body height [F(1, 304) = 5.97, *p* = 0.02, η^2^ = 0.02] and body mass [F(1, 304) = 7.23, *p* = 0.01, η^2^ = 0.02]. Boys also had significantly higher BMI values compared to girls [F(1, 304) = 5.45, *p* = 0.02, η^2^ = 0.02]. Girls had significantly lower waist circumference [F(1, 304) = 6.39, *p* = 0.01, η^2^ = 0.02] and lower waist-to-hip ratio values [F(1, 304) = 8.31, *p* < 0.001, η^2^ = 0.03]. Differences in hip circumference were not statistically significant (*p* = 0.27, η^2^ = 0.00), while body fat percentage (BF%) showed a trend toward higher values in girls without reaching statistical significance [F(1, 304) = 3.34, *p* = 0.07, η^2^ = 0.01]. Estimated VO_2_max did not significantly differ between sexes [F(1, 304) = 0.03, *p* = 0.86, η^2^ = 0.00], nor did total PA [F(1, 304) = 0.37, *p* = 0.54, η^2^ = 0.00]. In the domain of sedentary behavior, boys spent significantly more time watching television [F(1, 304) = 6.30, *p* = 0.01, η^2^ = 0.01], whereas differences in internet use were not statistically significant. Additionally, total physical activity (PAQ-C scores) did not differ significantly across the three screen-time categories (F(2,303) = 0.51, *p* = 0.604, η^2^ = 0.003). Bonferroni *post hoc* comparisons also showed no significant pairwise differences between screen-time groups (all *p* > 0.96).

**Table 3:** Results of analysis of variance for determining differences in morphological characteristics, physical fitness, physical activity, and screen time according to sex.

Variable	M	SD	M	SD	F	*p*	Partial Eta squared
	Boys (*N* = 143)		Girls (*N* = 163)		ANOVA		
Age	9.92	0.62	9.87	0.57	0.56	0.46	0.00
Body height (cm)	146.52^*^	8.19	144.24	8.14	5.97	0.02	0.02
Body weight (kg)	42.51^*^	12.95	38.86	10.76	7.23	0.01	0.02
Fat mass %	19.76	8.81	21.61	8.88	3.34	0.07	0.01
BMI	19.42^*^	4.47	18.31	3.89	5.45	0.02	0.02
WC (cm)	67.74	10.72	64.74^*^	10.00	6.39	0.01	0.02
HC (cm)	79.38	10.45	78.08	10.25	1.21	0.27	0.00
WHR	0.85	0.06	0.83^*^	0.06	8.31	0.00	0.03
VO_2_ max	41.12	2.18	41.16	1.82	0.03	0.86	0.00
Physical activity	2.99	0.73	2.94	0.72	0.37	0.54	0.01
TV time (hours)	1.23^*^	0.96	0.96	0.90	6.30	0.01	0.00
Internet time (hours)	1.41	1.04	1.31	1.14	0.63	0.43	0.00

M, mean; SD, standard deviation; Min, minimum values; ^*^denotes significant differences *p* < 0.05.

Table 4 presents the results of the two-way analysis of covariance (ANCOVA), which examined the effects of screen time categories and sex on cardiorespiratory capacity (VO_2_max), while controlling for total PA. A statistically significant main effect of screen time category on cardiorespiratory capacity (VO_2_max) was found, F(2, 299) = 6.40, *p* < 0.001, with an effect size of partial η^2^ = 0.04. Total PA as a covariate was also statistically significant (F = 4.56, *p* = 0.03), with an effect size of η^2^ = 0.02. No statistically significant main effect of sex was found (*p* = 0.58); however, a statistically significant interaction between sex and screen time category was identified, F(2, 299) = 4.47, *p* = 0.01, with an effect size of η^2^ = 0.03. The overall model explained 8% of the variance in VO_2_max (adjusted R^2^ = 0.08). Although statistically significant, the effects of screen time and the interaction between sex and screen time were associated with small effect sizes (partial η^2^ = 0.03–0.04).

**Table 4:** Results of the two-way analysis of covariance (ANCOVA) for determining the interaction effect of sex and screen time on cardiorespiratory fitness.

Source	Type III SS	df	MS	F	*p*	Partial Eta squared
Corrected model	91.67	6	15.27	4.08	0.00	0.08
Physical activity	17.07	1	17.07	4.56	0.03	0.02
Sex	1.17	1	1.17	0.31	0.58	0.00
Screen category	47.93	2	23.96	6.40	0.00	0.04
Sex ^*^screen category	33.49	2	16.75	4.47	0.01	0.03
Error	1,119.15	299	3.74			
Total	519,195.46	306				
Corrected total	1,210.77	305				

Adjusted estimated marginal means presented in Table 5 showed that children in the high screen-time category had lower VO_2_max values (M = 40.39 ± 0.24) compared to children in the low (M = 41.34 ± 0.22) and moderate screen-time categories (M = 41.37 ± 0.15), which showed nearly identical values. The difference between the high and low categories was approximately 0.98 mL·kg^−1^·min^−1^, indicating approximately 2.4% lower estimated VO_2_max values in the high screen-time group. Among boys, the observed differences ranged from 1.56 to 1.92 mL·kg^−1^·min^−1^, corresponding to approximately 4–5% lower VO_2_ max in the high screen-time group compared with the low and moderate screen-time groups.

**Table 5:** Adjusted estimated marginal means of VO_2_max by screen time category derived from the ANCOVA model (adjusted for total physical activity and sex).

Screen category	Mean	Std.error	95% Confidence interval	
			Lower bound	Upper bound
1 Low screen time	41.34	0.22	40.91	41.77
2 Average screen time	41.37	0.15	41.07	41.68
3 High screen time	40.39	0.24	39.92	40.87

**Figure 1:**
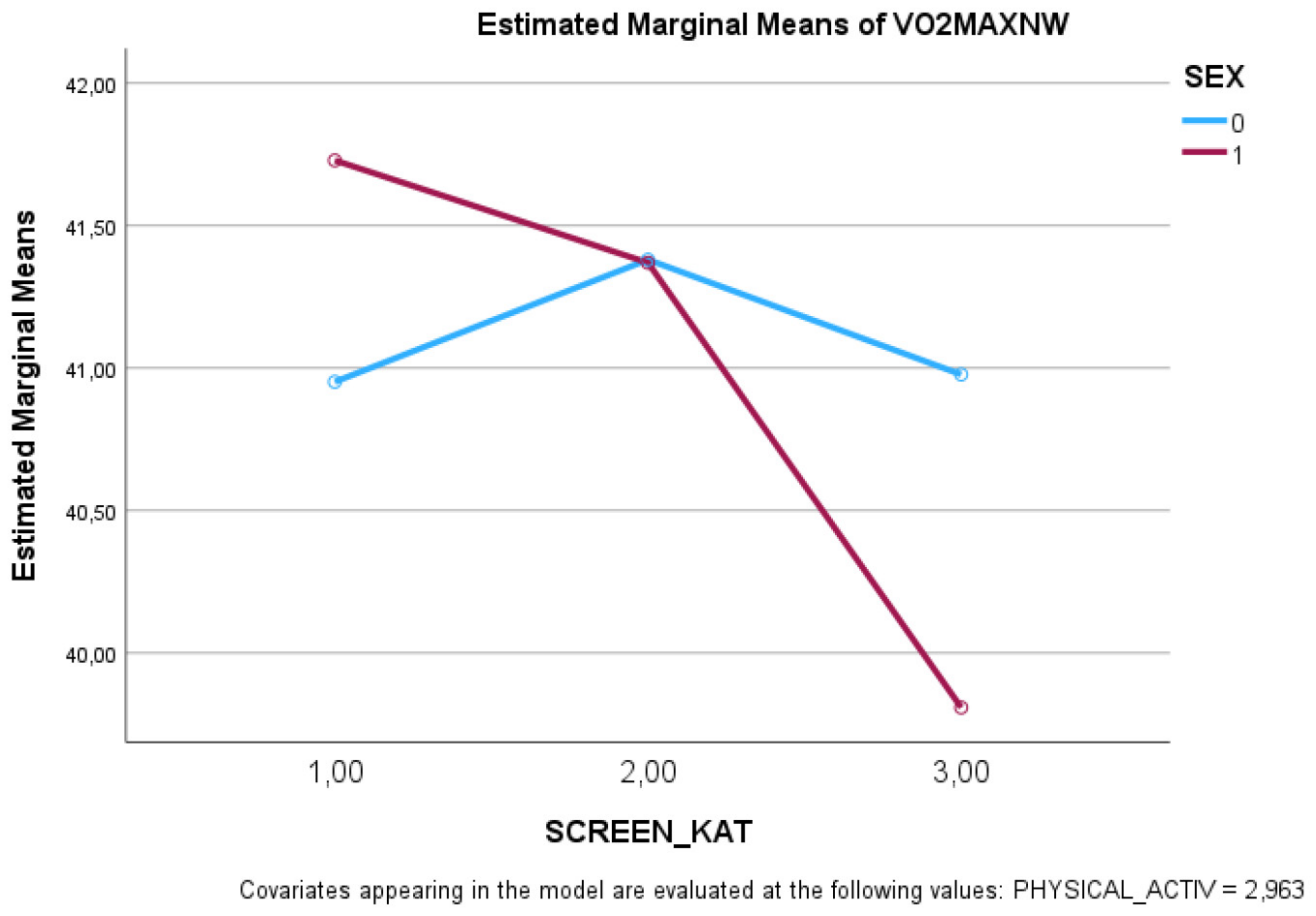
Comparisons of adjusted estimated VO_2_max means according to screen time categories within each sex, controlling for physical activity as a covariate.

The significant interaction between sex and screen time category was further examined using Bonferroni pairwise comparisons (Table 6). The interaction is illustrated in Figure 1. Among girls, no statistically significant differences in cardiorespiratory fitness were observed between screen time groups. By contrast, boys in the high screen-time group had significantly lower VO_2_max values compared to boys in the low screen-time group (mean difference = 1.92 ml·kg^−1^·min^−1^, *p* < 0.001) and the moderate screen-time group (mean difference = 1.56 ml·kg^−1^·min^−1^, *p* = 0.01). The interaction plot (Figure 1) showed non-parallel lines, visually confirming the presence of a significant interaction effect between sex and screen time. Diagnostic analyses indicated no influential observations in the ANCOVA model. The maximum Cook's distance was 0.061, while standardized residuals ranged from −2.47 to 2.89 and studentized residuals ranged from −2.52 to 2.95, all of which were within acceptable limits. Furthermore, sensitivity analyses excluding the three observations with the highest Cook's distance values produced virtually identical ANCOVA results, with both the main effect of screen time and the interaction between sex and screen time remaining statistically significant. These findings indicate that the reported results were robust and were not driven by a small number of influential observations.

**Table 6:** Bonferroni *post hoc* comparisons of estimated VO_2_max means according to screen time categories within each sex.

Pairwise comparisons							
Dependent variable: cardiorespiratory capacity							
Sex	(I) Screen category	(J) Screen category	Mean difference (I-J)	Std. error	*p*	95% confidence interval for difference	
						Lower bound	Upper bound
Girls	1	2	−0.43	0.35	0.68	−1.28	0.42
		3	−0.03	0.42	1.00	−1.03	0.98
	2	1	0.43	0.35	0.68	−0.42	1.28
		3	0.40	0.39	0.90	−0.53	1.34
	3	1	0.03	0.41	1.00	−0.98	1.03
		2	−0.40	0.39	0.90	−1.34	0.53
Boys	1	2	0.36	0.41	1.00	−0.64	1.36
		3	1.92^*^	0.50	0.00	0.72	3.13
	2	1	−0.36	0.41	1.00	−1.36	0.64
		3	1.56^*^	0.42	0.00	0.55	2.57
	3	1	−1.92^*^	0.50	0.00	−3.13	−0.72
		2	−1.56^*^	0.42	0.01	−2.57	−0.55

* Statistical significance set at *p* < 0.05; Mean difference, difference between group means; Std. Error, standard error; *p* values adjusted for multiple comparisons using the Bonferroni correction; CI lower, lower bound of the 95% confidence interval; CI upper, upper bound of the 95% confidence interval; Screen category: 1, 0–2 h/day (low), 2, 2–4 h/day (moderate), 3, >4 h/day (high).

## 4 Discussion

Based on the study aim, the relationship between screen time and cardiorespiratory fitness in school-aged children was examined while controlling for total PA and determining the presence of sex differences in this relationship. The main finding of the study indicates that a high level of screen time is associated with lower values of cardiorespiratory fitness, with this relationship being observed exclusively among boys. The obtained results may be interpreted in the context of current recommendations stating that children aged 5–17 years should not spend more than 2 h per day in recreational screen time (11, 30), although clearly defined limits for optimal screen time exposure still do not exist (31).

The results of the two-way ANCOVA further confirmed that the screen time category had a statistically significant effect on cardiorespiratory fitness, independent of PA level. Children in the high screen-time category had lower adjusted maximal oxygen uptake (VO_2_max) values compared to children in the low and moderate categories. These findings are consistent with numerous previous studies reporting a negative association between screen time and cardiorespiratory fitness (18, 32–38). However, some recent studies suggest that this relationship is not always statistically significant after controlling for other factors such as total PA or other components of physical fitness (8, 39).

It is particularly important to emphasize that differences between children in the low and moderate screen-time groups were not statistically significant, whereas significant differences were found between the high category and both lower categories. This pattern suggests a nonlinear relationship between screen time and cardiorespiratory fitness, whereby the negative effect primarily emerges at higher levels of screen exposure. In other words, moderate exposure to digital screens is not associated with reduced cardiorespiratory fitness, whereas excessive exposure may be associated with negative physiological consequences and measurable reductions in aerobic capacity. A difference of approximately 0.98 ml·k^−1^·min^−1^ represents about a 2–3% difference in VO_2_max, which may be of potential clinical relevance, particularly considering the well-established relationship between childhood cardiorespiratory fitness and long-term cardiometabolic health.

Although numerous studies confirm a negative relationship between sedentary behavior and cardiorespiratory fitness (40) and indicate that prolonged screen exposure may reduce cardiorespiratory fitness (16), results are not always consistent, especially when analyses control for children's total PA.

The obtained results are consistent with international studies showing that between 50% and 70% of school-aged children exceed the recommended limit of 2 h of recreational screen time per day. The findings are in agreement with previous studies (18, 41–44), while the proportion of children in the high screen-time category (21.6%) falls within the range reported in previous studies (20%−30%) (2, 45). These findings confirm the global trend of increasing sedentary behavior while simultaneously indicating that the observed population does not substantially differ from international patterns.

Regarding sex differences in morphological characteristics and physical fitness indicators, although boys demonstrated significantly higher body height, body mass, and BMI values, the effect sizes were small, suggesting limited practical relevance. No significant sex differences were found in VO_2_max or total physical activity. This finding is inconsistent with many previous studies reporting higher VO_2_max values and physical activity levels in boys (40, 46–49), but is consistent with more recent studies conducted in prepubertal children that found no significant sex differences in these variables (50, 51). Similar findings were reported by López-Gil et al. (10), who also found no significant sex differences in cardiorespiratory capacity. These findings suggest a possible absence of pronounced sexual dimorphism in cardiorespiratory fitness and PA levels during the prepubertal period. However, most previous studies have reported higher VO_2_max values in boys compared to girls (50, 52–54). The reasons for these differences are not fully understood, although greater muscle mass in boys is assumed to play an important role (55).

In the present study, no significant sex differences were observed in total physical activity, suggesting that physical activity does not explain the observed sex-specific association between screen time and cardiorespiratory fitness. This finding differs from many previous studies reporting that boys are generally more physically active than girls (40, 44, 56, 57). Importantly, boys were not significantly more likely than girls to belong to the high screen-time category, and total physical activity did not differ across screen-time groups. These findings suggest that the observed interaction between sex and screen time cannot be explained simply by greater overall physical activity or by a higher prevalence of excessive screen exposure among boys. Interestingly, boys spent significantly more time watching television than girls, although the effect size was small (η^2^ = 0.01), whereas no significant sex differences were observed in internet use. Similar findings were reported by Whiting et al. (2021), who found that boys were more likely to spend longer periods in front of screens than girls, consistent with earlier studies (40), although some studies have not confirmed sex differences in screen time (49).

Considering that screen-time categories in this study were defined according to total daily duration, the obtained results suggest that higher levels of total screen exposure, particularly within the high category, may play an important role in explaining the observed differences in cardiorespiratory fitness between boys and girls.

Several potential mechanisms may help explain why this association was observed primarily among boys. First, boys in the present sample spent significantly more time watching television than girls, suggesting a higher baseline exposure to this specific form of screen-based sedentary behavior. Second, a differential substitution effect may be involved, whereby screen time in boys could displace higher-intensity activities, such as vigorous outdoor or sports play, that are not fully captured by the general PAQ-C activity score, whereas girls' activity patterns may be less affected by this substitution. Third, sex-related differences in physiological adaptation to prolonged sedentary behavior during the prepubertal period may also contribute to the observed findings, potentially through differences in muscle mass development or cardiovascular maturation. These proposed mechanisms remain hypothetical and should be confirmed in future longitudinal studies using objective measures of physical activity, screen time, and physiological markers.

Although no statistically significant sex differences were identified in either cardiorespiratory fitness or total PA, a two-way analysis of covariance (ANCOVA) was applied to further examine whether differences in cardiorespiratory capacity existed while controlling for PA as a covariate. The use of ANCOVA was methodologically justified because total PA has been theoretically and empirically confirmed as one of the key predictors of cardiorespiratory fitness (58, 59). In addition, a genetic association between cardiorespiratory fitness and PA levels has also been identified (60), indicating their interrelationship at the biological level. Accordingly, individuals with higher levels of PA generally achieve higher VO_2_max values (61). After including PA as a covariate in the model, the results showed that sex alone did not have a statistically significant main effect on VO_2_max; however, a significant interaction between sex and screen time was identified. This finding indicates that the relationship between screen time and cardiorespiratory capacity differs according to sex. Specifically, among boys, a high level of screen time was significantly associated with lower cardiorespiratory fitness compared to the low and moderate categories, whereas among girls, such an association was not statistically significant.

Among boys, the observed differences were more pronounced (1.56–1.92 ml·kg^−1^·min^−1^), representing approximately a 4–5% difference in aerobic capacity. Although these absolute differences are relatively modest, they may still be clinically relevant given the established association between childhood cardiorespiratory fitness and future cardiometabolic health. Although these are relatively small absolute differences, such findings may still have meaningful implications from the perspective of children's functional capacity and health. This pattern suggests that the negative effect of screen time is not universal but sex-specific, further emphasizing the importance of considering interaction effects when analyzing the relationship between sedentary behavior and cardiorespiratory fitness.

The inclusion of total physical activity as a covariate enabled the independent association between screen time, sex, and cardiorespiratory fitness to be examined while accounting for variability attributable to overall physical activity. After adjustment for total physical activity, no significant main effect of sex on VO_2_max was observed; however, the interaction between sex and screen time remained statistically significant. This finding indicates that the association between screen time and cardiorespiratory fitness differs according to sex, independently of overall physical activity. Adjusting for total physical activity reduced variability attributable to differences in overall activity levels, allowing a more precise estimation of the relationship between screen time, sex, and cardiorespiratory fitness. Although the model explained a relatively small proportion of the variance in VO_2_max, this finding was expected given the complex and multifactorial nature of cardiorespiratory fitness in children, which is influenced by numerous biological, behavioral, and environmental factors.

The findings are consistent with previous studies indicating a negative association between excessive screen exposure and cardiorespiratory fitness even after adjustment for PA (62). Since the results of this study demonstrate that the association between screen time and VO_2_max remains significant even after controlling for total PA, the findings suggest that the association between screen time and cardiorespiratory fitness may be at least partly independent of overall physical activity levels. Similar findings have been reported in longitudinal studies showing that high levels of screen time predict lower cardiorespiratory fitness and less favorable cardiometabolic profiles (63, 64). Although the observed associations were statistically significant, their effect sizes were generally small and should therefore be interpreted with appropriate caution. Nevertheless, even modest differences in cardiorespiratory fitness at the population level may have meaningful public health implications, given the well-established association between childhood cardiorespiratory fitness and long-term cardiometabolic health.

### 4.1 Strengths and limitations

Despite the valuable findings of this study, several limitations should be considered when interpreting the results. First, the study was conducted using a cross-sectional design, which prevents causal conclusions regarding the relationship between screen time and cardiorespiratory fitness. Furthermore, screen time was assessed through self-report measures, which may be associated with recall bias or socially desirable responses. This method of assessment may therefore lead to inaccuracies in estimating the actual amount of time spent using digital devices. In addition, the questionnaire assessed overall daily screen time and did not distinguish between recreational and educational/school-related screen use or between different types of screen-based activities (e.g., television viewing, gaming, social media, or educational use). Consequently, the behavioral mechanisms underlying the observed sex-specific association could not be examined directly. Future studies should differentiate between these types of screen exposure to provide a more detailed understanding of their potential health effects.

In addition, several potentially important factors were not included in the study, such as biological maturation status, socioeconomic status, or dietary habits, all of which may act as additional predictors of cardiorespiratory fitness. Consequently, residual confounding by these and other unmeasured variables cannot be excluded, despite statistical adjustment for total physical activity. The relatively modest proportion of variance explained by the model (adjusted R^2^ = 0.08) is consistent with the multifactorial nature of cardiorespiratory fitness and the contribution of additional biological, behavioral, and environmental determinants that were not included in the present study.

Including these variables in future studies could contribute to a more comprehensive understanding of the relationship between sedentary behavior and cardiorespiratory fitness in children. Furthermore, future research should employ more objective methods for measuring screen time, such as digital applications or software for monitoring device usage, in order to improve the accuracy of the collected data. Despite these limitations, the present study also has several important scientific strengths. First, the study was conducted on a relatively large sample of school-aged children, allowing for a more reliable estimation of the relationship between screen time and cardiorespiratory fitness. A particular strength of the study is the application of analysis of covariance (ANCOVA), which controlled for the influence of total PA as an important predictor of cardiorespiratory fitness, thereby enabling a more precise estimation of the independent effect of screen time.

Additionally, interactions between sex and screen time were analyzed, providing deeper insight into the complexity of the relationship between sedentary behavior and cardiorespiratory fitness. In this way, new insights were obtained regarding possible behavioral and physiological differences between boys and girls. The findings of this study expand existing knowledge about the impact of the digital environment on children's health and provide new insights into sex-specific patterns in the association between screen time and cardiorespiratory fitness in school-aged children.

## 5 Practical implications

The results of this study have important practical implications for public health, the educational system, and the family environment. The identified association between screen time exceeding 4 h per day and lower cardiorespiratory fitness among boys highlights the importance of systematic monitoring and limitation of recreational screen exposure. Given that differences in cardiorespiratory capacity were observed already during childhood, it is important to promote a balanced relationship between digital device use and participation in PA, while ensuring sufficient time for active play and organized sports activities. For example, schools may establish school policies that encourage children to interrupt prolonged periods of sedentary behavior by integrating regular movement opportunities throughout the school day and by promoting participation in extracurricular physical activity programmes.

The findings also emphasize the importance of educating parents about the potential health consequences of excessive screen time. Public health programs and interventions aimed at reducing sedentary behavior should begin during the early school years and include recommendations to limit recreational screen time to less than 2 h per day, in accordance with international guidelines. Parents may also be encouraged to establish consistent household rules regarding recreational screen time and to model healthy screen-use behaviors while promoting regular participation in organized sports and outdoor recreational activities, particularly among boys, who showed the strongest association between screen time and cardiorespiratory fitness in the present study.

Finally, the identified sex-specific association between screen time and cardiorespiratory fitness suggests that future preventive strategies may benefit from considering differences in behavioral patterns between boys and girls, in order to more effectively adapt interventions to their specific needs.

## 6 Conclusion

Based on the findings of this cross-sectional study, higher levels of screen time were associated with lower cardiorespiratory fitness among school-aged children, with this association being particularly pronounced in boys. Analysis of covariance showed that the association between screen time and cardiorespiratory fitness remained statistically significant after adjustment for total physical activity, suggesting that this association is at least partly independent of overall physical activity. The findings further indicate that the association was primarily evident among children with high levels of screen exposure, whereas no significant differences were observed between the low and moderate screen-time groups.

In addition, the significant interaction between sex and screen time suggests a sex-specific pattern of association, whereby a significant association was observed among boys, whereas no such association was found among girls. Although these findings highlight the potential importance of limiting excessive recreational screen time while promoting regular physical activity, the cross-sectional design of the study does not allow causal inferences to be made. Future longitudinal studies are needed to clarify the direction of these associations and to determine whether reducing excessive recreational screen time is associated with improvements in cardiorespiratory fitness. Particular attention may be warranted for boys, who appear to represent a potentially more vulnerable group.

## Data Availability

The raw data supporting the conclusions of this article will be made available by the authors, without undue reservation.
